# Exercise of Varying Durations: No Acute Effects on Cognitive Performance in Adolescents

**DOI:** 10.3389/fnins.2018.00672

**Published:** 2018-09-27

**Authors:** Vera van den Berg, Emi Saliasi, Jelle Jolles, Renate H. M. de Groot, Mai J. M. Chinapaw, Amika S. Singh

**Affiliations:** ^1^Department of Public and Occupational Health, Amsterdam UMC, Vrije Universiteit Amsterdam and the Amsterdam Public Health Research Institute, Amsterdam, Netherlands; ^2^Faculty of Behavioral and Human Movement Sciences, Centre for Brain & Learning, LEARN! Institute, VU University Amsterdam, Amsterdam, Netherlands; ^3^Welten Institute - Research Centre for Learning, Teaching and Technology, Open University of the Netherlands, Heerlen, Netherlands; ^4^Department of Complex Genetics, School for Nutrition, Toxicology and Metabolism/Faculty of Health, Medicine and Life Sciences, Maastricht University, Maastricht, Netherlands

**Keywords:** physical activity, cognitive performance, selective attention, working memory, exercise duration, dose-response, children, adolescents

## Abstract

Participation in structured physical activity is assumed to have a positive effect on cognitive and academic performance. A single bout of moderate to vigorous exercise has been found to have a small acute positive effect on the cognitive performance of children and adolescents. However, the dose-response effects of exercise duration are largely unknown. Therefore, the current study examined the acute effects of moderate-to-vigorous exercise with a duration of either 10, 20, or 30 min on selective attention and working memory performance of young adolescents. One hundred and nineteen adolescents (11–14 years old) participated in a randomized, controlled crossover study. Adolescents were assigned to one of the three exercise durations, each paired with a sedentary control session of the same duration. Cognitive performance was measured before and immediately after the exercise and control condition. The Attention Network Test and n-back task were used to measure selective attention and working memory, respectively. There were no significant exercise effects on selective attention (i.e., alerting, orienting, or executive control) or working memory performance measured immediately after the exercise bouts. Furthermore, there were no differential effects of exercise duration. In sum, acute exercise bouts with a duration of 10, 20, or 30 min did not improve, but neither deteriorate cognitive performance of young adolescents compared to a sedentary control condition.

## Introduction

The maturation of the adolescent brain is guided by an interaction between genetic and environmental factors (Rosenzweig, 2003; Lenrootand Giedd, 2008). Among these factors, physical activity (PA) has been well studied, in particular because its potential beneficial effects on cognitive functioning and academic achievement. Two systematic reviews concluded that *overall*, single bouts of PA have small positive acute effects on cognitive performance of children and adolescents (Donnelly et al., 2016; Ludyga et al., 2016). In addition, a recent meta-analysis concluded that PA can have acute positive effects on attention and inhibition in pre-adolescent children (de Greeff et al., 2018). Besides evidence on the acute effects, meta-analyses of longitudinal studies have shown that engaging in structured PA sessions can have a neutral or positive effect on cognitive functioning in children, and certainly does not harm children's performance (e.g., Li et al., 2017; Watson et al., 2017; de Greeff et al., 2018).

Despite the positive effects of PA on mental (Biddle and Asare, 2011) and physical health (Janssen and Leblanc, 2010), and its assumed effect on cognitive functioning, there is ample evidence that many children and adolescents do not meet PA guidelines (WHO, 2010; Health Council of the Netherlands, 2017). Schools are seen as the most appropriate setting to enforce structural opportunities to increase PA levels in children and adolescents as they spend a substantial amount of their time at school (WHO, 2010, 2014; Webster et al., 2015). However, time constraints are a frequently mentioned barrier that hinders implementation of PA in schools (e.g., Howie et al., 2014b; McMullen et al., 2014; Naylor et al., 2015; Stylianou et al., 2015; van den Berg et al., 2017). Therefore, teachers have indicated that it would only be feasible to implement short PA bouts in the school curriculum, with a maximum of 5 (Howie et al., 2014b) or 10 min per session (van den Berg et al., 2017).

Although studies have consistently shown that the intensity of acute PA needs to be of at least moderate to vigorous intensity to gain most cognitive benefits (McMorris and Hale, 2012; Peruyero et al., 2017), the optimal duration of acute PA is still unclear and needs further investigation (Janssen et al., 2014b; Verburgh et al., 2014; Donnelly et al., 2016). Previous studies have shown that acute exercise bouts with a duration of 30 or more minutes can improve children's and adolescent's performance in inhibition and shifting (Ellemberg and St-Louis-Deschênes, 2010; Chen et al., 2014), working memory (Pontifex et al., 2009; Chen et al., 2014), selective attention (Gallotta et al., 2012), free-recall memory (Pesce et al., 2009), planning (Pirrie and Lodewyk, 2012), and executive attention (Kubesh et al., 2009). However, studies by Pirrie and Lodwyk (2012, information processing and selective attention) and (Kubesh et al. (2009), working memory and cognitive flexibility) reported no effects. The effects of a medium exercise duration (i.e., 20 min) on cognition are also inconclusive, with some studies showing improved performance in inhibitory control (Hillman et al., 2009; Drollette et al., 2012, 2014), comprehension (Hillman et al., 2009), and selective attention (depending on time of the day; Altenburg et al., 2016), and others showing no effects on inhibitory control (Stroth et al., 2009), working memory (Drollette et al., 2012), and broad measures of executive functioning (Howie et al., 2015). Also, the effects of exercise of a shorter duration of 10–15 min are inconclusive: beneficial effects have been reported on selective attention (Budde et al., 2008; Niemann et al., 2013; Janssen et al., 2014a), working memory (depending on exercise intensity and performance level) (Budde et al., 2010), as well as on broad measures of executive functioning (Cooper et al., 2012, 2016; Benzing et al., 2016), while no effects on selective attention and information processing (van den Berg et al., 2016), visuo-spatial memory and general psychomotor speed (Cooper et al., 2016), sustained attention (Wilson et al., 2016), and executive functioning (Howie et al., 2015) have been reported. Studies examining the effects of 5-min exercise sessions found no effects (Kubesh et al., 2009; Howie et al., 2014a, 2015). In sum, the evidence on the acute effects of relatively short, medium, and long exercise bouts on cognitive performance is inconclusive and the differences in cognitive outcome measures across studies make it particularly difficult to compare the effects of exercise bouts with different durations with each other. Therefore, dose-response studies are needed to be able to elucidate the acute effects of exercise duration on cognitive performance.

To date, only few studies investigated the acute dose-response effects of exercise duration on cognitive performance in children and adolescents. Two studies of Howie and colleagues (Howie et al., 2014a, 2015) investigated whether the cognitive performance of children (aged 9–12 years) differed after 5, 10, and 20 min of moderate to vigorous classroom-based exercise compared to 10 min of sedentary activities (i.e., listening to a lesson about exercise science). The authors reported higher math fluency scores after 10 and 20 min of exercise compared to the sedentary condition (Howie et al., 2015), and improved on-task behavior after 10 min, but not after 5 and 20 min of exercise (Howie et al., 2014a). While these studies investigated the effect of different exercise durations, the authors conducted separate analyses and did not compare the effects of 5, 10, and 20 min exercise with each other. Recently, two studies in young male adults (20–23 years old) examined the dose-response relation between exercise duration and cognitive performance on a Color-Word Stroop task (Chang et al., 2015; Tsukamoto et al., 2017). Chang and colleagues found that 20 min moderate intensity exercise on a cycle ergometer resulted in larger improvements in cognitive performance than 10 or 45 min of exercise (Chang et al., 2015). In contrast, Tsukamoto and colleagues reported no difference in the positive effects of 10, 20, or 40 min moderate intensity cycle ergometer exercise on cognitive performance (Tsukamoto et al., 2017).

In the current study, we examined the dose-response effects of exercise duration (10, 20, or 30 min) on selective attention and working memory of young adolescents (11–14 years). We conducted a randomized controlled cross-over study in the school setting and assessed effects on attention and working memory as these cognitive functions are associated with academic achievement (Stevens and Bavelier, 2012; van der Ven et al., 2013). Selective attention is defined as “the differential processing of simultaneous sources of information” (Johnston and Dark, 1986, p. 44). In other words, it determines which stimuli are relevant and which are irrelevant and should be suppressed (Ellemberg and St-Louis-Deschênes, 2010). Working memory is a cognitive function with limited capacity that allows individuals to temporarily store and actively manipulate information over a brief period of time (Baddeley, 2003). Based on the results of earlier studies, we hypothesize that moderate to vigorous exercise bouts of different durations will have a neutral or positive acute effect on selective attention and working memory performance of young adolescents.

## Methods

### Sample size calculation

An independent statistician performed a sample size calculation based on the effect size (partial η^2^ = 0.12) of an earlier study using a similar research design and cognitive tasks (Drollette et al., 2012). A sample of ~62 adolescents was needed to detect within-subjects effects (i.e., exercise effects on cognition), and ~105 participants to detect within-between interaction effects (i.e., differential effects of exercise duration) with 80% power, 2-sided testing at α = 0.05.

### Participants

We invited a convenience sample of three elementary schools and one secondary school to participate with all apparently healthy adolescents attending the last grade of elementary school (11–12 years) or the first grade of secondary school (12–13 years). First, we provided detailed information on the study to the school staff. After obtaining their consent, we provided adolescents and their parents with written information about the procedure and the scope of the study. Written informed consent was obtained from a parent/caregiver, and adolescents who were 12 years or older. Adolescents with a confirmed medical condition that could affect memory or concentration (e.g., ADHD, epilepsy) were identified by the school staff and were not included in the statistical analyses. Adolescents received a small present for their participation after the study. The Medical Ethical Committee of the VU University Medical Center in Amsterdam, the Netherlands, concluded that the study does not fall within the scope of the Medical Research Involving Human Subjects Act and approved the study protocol [2014.363].

### Design and randomization

We conducted a randomized controlled trial with a crossover design, including two within- and one between-subjects variables. The first within-subjects variable was “intervention session”: all adolescents performed one control and one exercise session of the same duration (i.e., either 10, 20, or 30 min), thereby acting as their own control. The second within-subjects variable was “test”: we conducted cognitive tests before (*pretest*) and after (*posttest*) the control and exercise session, to control for intra-individual differences in test performance. The between-subject variable was the “duration” of the exercise/control session: 10, 20, or 30 min. The order of the control and exercise session was counterbalanced, i.e., half of the adolescents started with the control session, and the other half with the exercise session.

We applied a block-random selection procedure to determine duration (i.e., 10, 20, or 30 min) and sequence (i.e., order of exercise and control session) using two online software programs (http://www.randomizer.org/form.htm and http://www.graphpad.com/quickcalcs/randomize2/). We stratified by sex in the randomization procedure.

### Procedure

**Figure 1:**
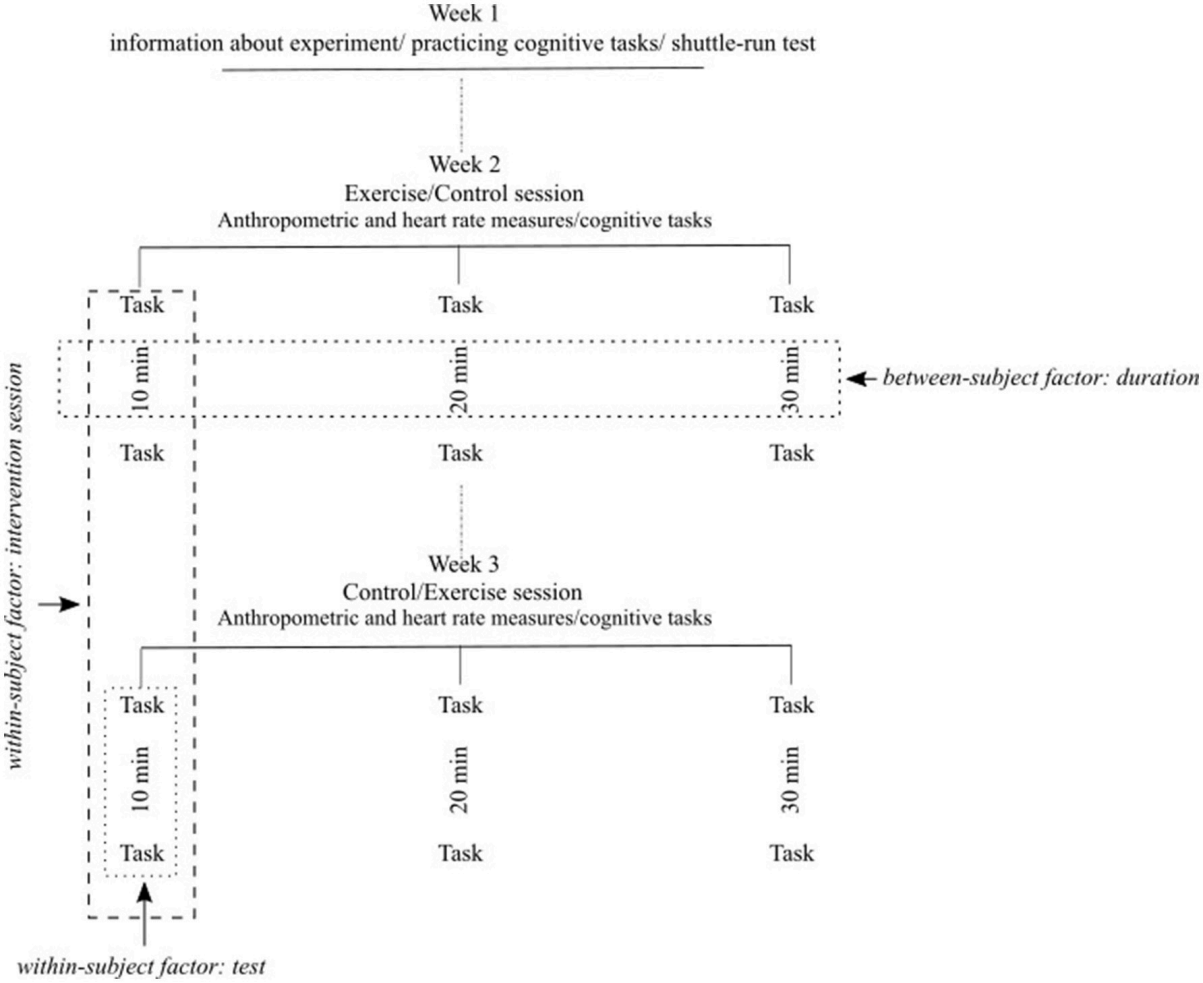
Schematic representation of the experimental design.

We visited the schools on four separate occasions within a period of 3 weeks (see Figure 1). The first and the second visit were generally scheduled within the same week. The first visit consisted of a familiarization session in which adolescents received detailed information on the experiment and practiced with the two cognitive tasks (see section Cognitive Measures). During the second visit, we assessed their maximum heart rate and fitness by means of a Shuttle Run test. The third and fourth visit consisted of the experimental days, which were scheduled 1 week apart at the same time of the day (between 08:00 and 11:45 a.m.). We asked the adolescents to keep their bedtime, breakfast, and transport mode to school similar before each experimental day. We invited groups of four to six students to the testing location, which was a private area within the school. Each day had the same standardized routine: (1) all adolescents practiced with the two cognitive tasks and self-reported their sleep, breakfast, mode, and duration of transportation to school; (2) in the exercise session we measured height and weight of the adolescents and provided them with a Polar RS800cx heart rate monitor. After adolescents laid on their back comfortably for 5 min, with legs and arms positioned along the body, the resting heart rate was measured to calculate the heart rate zone corresponding to moderate to vigorous intensity (see section Exercise Bout); (3) adolescents performed two cognitive tasks on a laptop (pretest); (4) during the exercise session adolescents cycled for 10, 20, or 30 min, whereas during the control session they worked on educational materials (e.g., puzzles, questionnaires, worksheets) seated for either 10, 20, or 30 min; (5) adolescents performed the two cognitive tasks again (posttest) on the same laptop.

### Exercise bout

The exercise bout followed a bicycle ergometer protocol. The adolescents biked at moderate to vigorous intensity for a duration of 10, 20, or 30 min. The first minute and a half served as warming-up [workload = 0 kilopond (Kp)]. After this period, the workload increased until adolescents biked within the predetermined boundaries of their moderate to vigorous intensity level. The maximum heart rate and resting heart rate were used to calculate the lower (40%) and upper (60%) boundary of the heart rate reserve, corresponding to a moderate to vigorous intensity level of exercise. The boundaries were calculated as follows: 40% = [(maximal heart rate – resting heart rate) ^*^ (40/100) + resting heart rate]; 60% = [(maximal heart rate – resting heart rate) ^*^ (60/100) + resting heart rate] (ACSM, 2010). Adolescents who did not reach the specified heart rate zone were excluded from the data analyses (i.e., if the mean exercise intensity was below 40% or above 60% of the heart rate reserve). To facilitate biking in a steady state manner, the number of flywheel revolutions per minute was matched to a metronome with 120–160 beats per minute. The last minute served as cooling down, in which the flywheel revolutions per minute were progressively reduced (workload = 0 Kp).

## Measures and measurement instruments

### Anthropometrics

Height (cm) and weight (kg) were measured in sport clothing using a Seca weighing scale (Seca Instruments, Frankfurt, Germany) and a Leicester Height Measure Mk II (Harlow Healthcare, UK). Body Mass Index (BMI) was calculated by dividing weight (kg) by height squared (m).

### Shuttle run test

We administered a Shuttle Run test to assess the maximum heart rate and cardiovascular fitness (VO_2_ max) of the adolescents. All participants wore a Polar H7 heart rate monitor that was connected to the Polar Team App (Polar Electro Oy, Finland), in which the heart rate data was stored. The test was performed during a regular physical education lesson, under the supervision of the physical education teacher. All adolescents were familiar with this test and were encouraged by their teacher and the research team to exert maximum performance.

Due to the dimensions of the sports halls, students in the elementary schools performed an 18 m instead of 20 m Shuttle Run, while secondary school students performed the standard 20 m test. The test had an initial running speed of 8.0 km/h that progressively increased with 0.5 km/h in 1 min stages in the 20 m test. This corresponded with an initial running speed of 7.2 km/h increasing with 0.45 km/h in 1 min stages in the 18 m test. We recorded the highest completed stage with an accuracy of half a stage and calculated VO_2_max (ml/kg/min) (Léger et al., 1988).

### Cognitive measures

We used two computerized cognitive tasks: the Attention Network Test (ANT) and the n-back task. Both tasks have been shown to have optimal criterion validity, good statistical dependencies and adequate factorial structure, suggesting that these tasks are valid measures of cognitive performance in children (Forns et al., 2014). The tasks were programmed using E-Prime 1.2 software (Psychology Software Tools, Pittsburgh, PA), which was also used for stimulus generation and response registration. To minimize interference during the tasks, a maximum of two adolescents were seated at one working desk, facing each other supervised by a member of the research team. We asked them to work quietly and individually and to focus on their task the entire time. The order in which the cognitive tasks were performed was randomized and counterbalanced between participants (first ANT and then n-back, or vice versa).

### Practice trials

During the familiarization session, the adolescents practiced 57 trials (nine trials with feedback) of the ANT and 150 trials (60 trials with feedback) of the n-back task. Given the complexity of the current n-back task, we incorporated a loop, which allowed them to repeat any part of the task (instructions or task blocks) until the task was fully understood. In addition, they made a few practice trials of both tasks at the start of each experimental day.

### Attention network test

We used the short-version (Fan et al., 2007) of the ANT (Fan et al., 2002) to assess three attentional networks: alerting (i.e., achieving and maintaining an alert state), orienting (i.e., selection of information from sensory input), and conflict/executive control (i.e., resolving conflict among responses). The stimuli of this task were sets of five horizontal black arrows presented on a white background. The middle arrow pointing either to the left or to the right was the target, flanked by two lateral arrows on the left and the right. The flanker arrows pointed either in the same direction of the target arrow (congruent flanker condition: >>>>> or < < < < <) or in the opposite direction of the target arrow (incongruent flanker condition: >> < >> or < < > < <). We instructed the adolescents to respond as fast and as accurately as possible by pressing the left mouse button if the target arrow was pointing to the left and by pressing the right mouse button if the target arrow was pointing to the right. Adolescents were asked to focus on the fixation cross that was presented in the middle of the screen. A warning cue in the form of an asterisk sign (^*^) appeared in 66.7% of the trials for a duration of 200 ms in the center (“center cue” condition), or above or below the fixation cross (“spatial cue” condition), while being absent in the remaining trials (“no cue” condition). Details on the task parameters can be found elsewhere (Fan et al., 2007).

The total task lasted ~12 min and consisted of three blocks of 48 trials, with 1 min breaks between the blocks. We calculated accuracy rates (proportion of correct responses) and mean reaction times of the correct responses for the three attentional networks by the following formulas: Alerting = (Score_no cue – Score_center cue); Orienting = (Score_center cue – Score_spatial cue); Conflict/executive control = (Score_incongruent – Score_congruent) (Fan et al., 2007). Responses with reaction times faster than 200 ms were considered as incorrect (Fan et al., 2007). For the alerting and orienting scores, a larger value for the difference in reaction time and a larger negative value for the difference in accuracy means better performance. For the conflict/executive control score, a smaller value for the difference in reaction time and a smaller negative value for the difference in accuracy means better performance. The task was downloaded from the website of the Sackler Institute for Developmental Psychobiology (www.sacklerinstitute.org/cornell/assays_and_tools/).

### n-back task

We assessed working memory using a visual n-back task. After the task instructions, a continuous stream of letters (consonants displayed in 40 points Arial) was presented. The letters appeared one by one in the middle of the screen for a duration of 500 ms. The time between the stimuli varied randomly between 1,000 and 2,000 ms. The distance between the adolescent and the screen was ~65 cm. The visual angle of the stimuli was ~1.25°.

The task had three load conditions: the 0-back, 1-back, and 2-back load. In the 0-back load, the target was the letter “X.” In the 1-back load, the target was any letter identical to the letter presented in the last trial preceding it. In the 2-back load, the target was any letter identical to the letter presented two trials preceding it. We asked the adolescents to respond as fast and as accurately as possible by manually pressing a “green button” for targets and a “red button” for non-targets. For half of the adolescents, the green and the red button corresponded, respectively, to the key 1 and 2 on the left side of the keyboard. For the other half, these buttons were reversed.

The total task contained three blocks of 60 trials each and lasted ~10 min. Each block contained one load condition and the order of blocks was semi-randomized and counterbalanced between participants. Adolescents could differentiate the load of the block only through the instructions that were displayed on screen between the blocks. In each block, targets occurred randomly in ~37% of the trials. We calculated accuracy rates (proportion of correct responses) and mean reaction times of the correct responses for each load condition. Responses faster than 200 ms were considered as incorrect. Faster reaction times and higher accuracy rates indicate better working memory performance.

### Data analysis

All analyses were performed in the SPSS version 22.0 (IBM Corp. Released 2013. Armonk, NY: IBM Corp.). We examined differences in demographic measures between the 10-, 20-, and 30-min exercise groups by means of univariate ANOVA (see Table 1). For both cognitive tasks, reaction time and accuracy scores were separately analyzed by means of repeated measures (RM) ANOVAs, with intervention session (exercise vs. control) and test (pretest and posttest) as within-subjects factors and duration (10, 20, and 30 min) as between-subjects factor. In addition, the factor load (0-, 1-, and 2-back) was entered as a within-subject factor for the analyses of the n-back task. Baseline reaction times for the n-back task and the ANT conflict/executive control score differed significantly between the exercise and control condition. Therefore, we included the pretest score as covariate in the respective RM ANOVA models. We report interactions between the factors intervention session, test, and duration in the Results section. Estimated effect sizes are reported using eta-squares (?^2^). Statistical significance was set at *p* < 0.05.

**Table 1:** Baseline characteristics of the total study sample, as well as each of the subgroups according to the duration of each intervention session (means and SD).

	**Selective attention (ANT)**								**Working memory (n-back task)**							
	**Total sample**		**10 min**		**20 min**		**30 min**		**Total sample**		**10 min**		**20 min**		**30 min**	
N (included/excluded)	99	20	34	8	30	8	35	4	92	27	30	12	28	10	34	5
Age (years) (included|excluded)	12.3 (0.6)	12.6 (0.6)	12.2 (0.7)		12.4 (0.7)		12.4 (0.5)		12.3 (0.6)	12.6 (0.7)	12.3 (0.6)		12.3 (0.6)		12.3 (0.5)	
Sex (n; male/female) (included|excluded)	47/52	10/10	17/17		13/17		17/18		42/50	16/11	14/16		12/16		16/18	
BMI^a^ (kg/length^2^)	17.9 (2.5)		17.8 (2.6)		17.5 (1.9)		18.2 (2.7)		17.7 (2.5)		18.0 (2.7)		17.4 (1.9)		18.1 (2.8)	
VO_2_ max score^b^ (ml/kg/min)	47.6 (4.9)		47.7 (4.8)		47.5 (5.7)		47.7 (4.4)		47.7 (4.7)		47.4 (4.8)		47.9 (4.9)		47.8 (4.5)	
Maximum HR (beats per minute)	207.0 (8.2)		208.8 (6.7)		206.4 (9.0)		205.6 (8.7)		206.9 (8.3)		208.8 (7.0)		206.7 (8.8)		205.5 (8.9)	
Resting HR (beats per minute)	73.3 (9.8)		73.9 (9.0)		74.7 (8.1)		71.5 (11.6)		73.5 (9.4)		73.9 (7.9)		75.3 (7.7)		71.6 (10.6)	
Average HR exercise (beats per minute)	134.6 (7.6)		132.9 (7.7)*		137.0 (6.4)		134.8 (8.1)		134.5 (7.7)		131.8 (7.9)*		137.2 (6.5)		134.8 (7.8)	
40% HRR (beats per minute)	126.7 (7.7)		127.7 (6.9)		127.4 (7.2)		125.1 (8.8)		126.8 (7.6)		127.7 (7.2)		127.9 (6.8)		125.2 (8.5)	
60% HRR (beats per minute)	153.4 (7.3)		154.6 (6.3)		153.7 (7.5)		151.9 (8.1)		153.4 (7.3)		154.6 (6.7)		154.1 (7.1)		151.9 (8.0)	

Descriptives are rounded to the first decimal. ANT, ^a^BMI values are based on 89 participant [10 min (31), 20 min (24) and 30 min (34)]; ^b^VO_2_ max values are based on 93 participants [10 min (33), 20 min (29) and 30 min (31)]; n-back: ^a^BMI values are based on 83 participant [10 min (27), 20 min (23) and 30 min (33)]; ^2^VO_2_ max values are based on 85 participants [10 min (29), 20 min (27), and 30 min (29)]. *Significant group difference in average HR during exercise, HR in the 10 min group is lower than HR in the 20 min.

Adolescents with accuracy rates lower than chance level, indicating that they did not appropriately understood or followed the task instructions, were excluded from the respective analysis. For the n-back task, we only excluded adolescents with a lower accuracy rate than chance level in the 0-back and 1-back loads due to the difficulty of the 2-back load condition for which lower accuracy scores can be expected.

### Between-tests timing

We aimed to assess the differential effect of exercise duration on cognitive performance. The groups did not only differ in terms of exercise duration, but also in terms of the timing between the pre- and posttest (i.e., between-tests timing). In order to exclude any possible influence of the between-tests timing on the effect of exercise on reaction time and accuracy, we additionally examined the interaction between test (pre- and post-test) and duration for each cognitive task.

## Results

### Participants

A total of 119 students participated. Data from 99 adolescents in the ANT and 92 adolescents in the n-back task were included in the statistical analyses (see Table 1). One adolescent achieved accuracy scores below chance level in the ANT and seven adolescents performed below chance level in the n-back task (0-back and 1-back). Data from 17 adolescents were incomplete [i.e., participated in only one test session (*n* = 15)] or data was lost due to technical problems [n = 2, one in the ANT and one in the n-back task]. Two adolescents were diagnosed with a medical condition and one adolescent exercised at a mean exercise intensity below 40% HRR.

### Descriptive characteristics

Characteristics of the total sample and the subgroups according to exercise duration are presented in Table 1. Adolescents in the 10, 20, and 30 min duration had similar age, sex ratio, BMI, VO_2_max, maximal HR, resting HR, 40% HRR, and 60% HRR values. Adolescents in the 10 min exercise group had lower average HR scores within the moderate to vigorous intensity zone than those in the 20 min group but not the 30 min group, whereas average HR in the 20 and 30 min group was similar.

### Cognitive performance

#### ANT

For all three attentional networks of the ANT, accuracy rates were not significantly different between the exercise and control condition, or between the 10-, 20-, and 30-min exercise groups. Likewise, reaction time was not significantly different between the exercise and control condition for alerting and orienting, nor for conflict/executive control after controlling for pretest score. We found no differences in reaction time performance between the 10-, 20-, or 30-min exercise groups. Pre- and post-test scores and F-statistics of the RM ANOVA models can be found in Tables 2, 3, respectively. There were no interactions between the factors test and duration, indicating that the results were not influenced by the time between the tests.

**Table 2:** ANT data: pre- and posttest scores in the control and exercise condition (means, standard errors and 95% confidence intervals).

	**Alerting**		**Orienting**		**Conflict/Executive control**	
	**Control**	**Exercise**	**Control**	**Exercise**	**Control**	**Exercise**
**REACTION TIME (ms)**						
Pretest	19.0 (2.8) [13.5; 24.5]	20.4 (2.4) [15.6; 25.2]	63.2 (2.9) [57.3; 69.1]	59.9 (2.5) [54.9; 64.9]	78.3 (3.3) [71.7; 84.9]	86.8 (2.9) [81.1; 92.6]
Posttest	22.3 (2.8) [16.8; 27.7]	23.0 (2.6) [17.7; 28.2]	57.4 (3.1) [51.3; 63.6]	60.4 (2.9) [54.7; 66.1]	72.0 (3.3) [65.5; 78.5]	70.1 (2.8) [64.6; 75.6]
**ACCURACY (%)**						
Pretest	1.1 (0.6) [−0.1; 2.2]	1.6 (0.4) [0.8; 2.5]	−2.2 (0.5) [−3.2; −1.1]	−1.9 (0.5) [−2.9; −0.9]	−7.7 (0.9) [−9.5; −5.9]	−7.8 (0.9) [−9.5; −6.0]
Posttest	1.1 (0.6) [0.0; 2.2]	1.7 (0.7) [0.3; 3.0]	−2.0 (0.5) [−3.0; −1.1]	−3.2 (0.5) [−4.2; −2.3]	−8.3 (0.7) [−9.8; −6.9]	−7.5 (0.9) [−9.2; −5.7]

**Table 3:** F-statistics of the RM ANOVA model for the ANT data (reaction time and accuracy).

**ANT**	**Reaction time**			**Accuracy**		
***RM ANOVA: factor interactions***	**Statistics**			**Statistics**		
	***F* (df1, df2)**	***p***	**η^2^**	***F* (df1, df2)**	***p***	**η^2^**
**ALERTING**						
Intervention session*test	0.023 (1,96)	0.880	0.000	0.000 (1,96)	0.987	0.000
Intervention session*test* duration	0.133 (2,96)	0.876	0.003	0.090 (2,96)	0.914	0.002
**ORIENTING**						
Intervention session*test	1.892 (1,96)	0.172	0.019	2.586 (1,96)	0.111	0.026
Intervention session*test* duration	0.155 (2,96)	0.856	0.003	0.777 (2,96)	0.463	0.016
**CONFLICT/EXECUTIVE CONTROL**						
Intervention session*test	0.616 (1,96)	0.434	0.007	1.397 (1,96)	0.240	0.014
Intervention session*test* duration	0.463 (2,96)	0.631	0.010	0.247 (2,96)	0.782	0.005

#### n-back

For accuracy, we found no significant differences between the exercise and control condition or between the 10-, 20-, or 30-min exercise groups. Likewise, after controlling for pretest scores, there were no significant differences between the exercise and control condition for reaction time performance, nor between the 10-, 20-, and 30-min exercise groups. Pre- and post-test scores and F-statistics of the RM ANOVA can be found in Tables 4, 5. In line with the ANT data, we observed no significant between-test time differences.

**Table 4:** n-back data: pre- and posttest scores in the control and exercise session (means, standard errors, and 95% confidence intervals).

	**Reaction time (ms)**		**Accuracy (%)**	
	**Control**	**Exercise**	**Control**	**Exercise**
Pretest	501.9 (8.4) [485.3; 518.5]	495.6 (7.5) [480.7; 510.5]	86.3 (0.8) [84.8; 87.8]	85.7 (0.8) [84.2; 87.3]
Posttest	497.0 (8.0) [481.1; 512.8]	501.4 (8.3) [484.8; 517.9]	84.7 (0.9) [82.8; 86.5]	84.9 (0.8) [83.2; 86.6]

**Table 5:** F-statistics of the RM ANOVA model for the n-back data (reaction time and accuracy).

**n-back**	**Reaction time**			**Accuracy**		
**RM ANOVA: factor interactions**	**Statistics**			**Statistics**		
	***F* (df1, df2)**	***p***	**η^2^**	***F* (df1, df2)**	***p***	**η^2^**
Intervention session*test	2.478 (1,89)	0.119	0.028	1.398 (1,89)	0.240	0.015
Intervention session*test* duration	2.205 (2,89)	0.116	0.048	0.749 (2,89)	0.476	0.017
Intervention session*test*load	0.627 (2,178)	0.536	0.007	0.888 (2,178)	0.413	0.010
Intervention session*test*load*duration	1.037 (4,178)	0.390	0.023	0.969 (4,178)	0.426	0.021

## Discussion

This study investigated the acute effects of 10, 20, and 30 min of moderate to vigorous intensity exercise on selective attention and working memory performance in 11–14 years old adolescents. In addition, we explored possible dose-response effects of exercise duration on cognitive performance.

We found no acute effects of exercise on selective attention and working memory performance and no differential effects of exercise duration, measured immediately after the exercise bouts.

Our results are in line with some earlier studies that neither found acute effects on selective attention after long (45 min; Pirrie and Lodewyk, 2012), medium (20 min; Stroth et al., 2009), or short (12 min; van den Berg et al., 2016) bouts of exercise. Other studies, however, did report positive effects on selective attention performance. For example, Budde et al. (2008), Gallotta et al. (2012), Niemann et al. (2013), and Janssen et al. (2014a) reported acute effects of single exercise bouts on selective attention in children and adolescents. A difference between above-mentioned studies and our study was the administration of a paper-and-pencil task (d2 test of attention) vs. a computerized Flanker task. However, the use of a different cognitive task might not fully explain the differences in the results, as our findings are also inconsistent with the results of studies that used comparable computerized Flanker tasks. The studies that assessed the acute effects of exercise with comparable Flanker tasks reported positive effects on children's and adolescent's reaction time (Kubesh et al., 2009; Chen et al., 2014) and accuracy scores (Hillman et al., 2009; Drollette et al., 2012). In contrast to our study, in which cognitive performance was measured immediately after the cessation of exercise, cognitive performance in the studies of Hillman et al. (2009), Kubesh et al. (2009), Drollette et al. (2012), Niemann et al. (2013), and Chen et al. (2014) was measured with a delay of ~5–38 min after the exercise session ended. All of the before mentioned studies reported positive effects of the exercise bouts on selective attention. Chang et al. (2012) reported in their meta-analysis that acute exercise effects on cognition are largest when cognitive tests are assessed 11–20 min after the exercise bout (Chang et al., 2012). Although we found no acute effects of exercise, it might be that exercise related effects on cognitive performance exist, but only become detectable sometime after cessation of the exercise bout. The timing of the cognitive task administration is therefore an important factor to consider in future “exercise-cognition” research. We recommend future research to gain more insight in the timing of the posttest measurements, for example by comparing children's cognitive performance immediately as well as with a delay after a single exercise bout. In addition, it would be interesting to include multiple follow-up measures or to compare effects of different posttest timings (e.g., after 10, 45, and 60 min) to see how long potential exercise-related effects remain. This type of research has however a considerable participant burden reducing the feasibility in the school setting. Another potential reason for the differences in results might be the differences with regard to the control conditions. For example, Hillman et al. (2009) and Drollette et al. (2012) used passive control conditions, i.e., seated rest in which children performed no activities, whereas the children in the control condition in our study were working on school-related tasks, i.e., more cognitively engaging activities. It has been hypothesized that performing any cognitive activities during the control condition, such as watching an educational video or reading a book, may cause acute effects in cognitive performance which may be absent in case of a completely passive control condition in which children are not allowed to do anything (Best, 2010). In this respect, there could have been too little contrast between the exercise and control condition in our study to detect subtle exercise related effects. Future research with various sedentary control conditions is needed to further explore this issue. Lastly, inconsistencies in results between our and other studies might be attributed to differences in the age of the participants, i.e., 11–14 years old adolescents in our study vs. 8–11 years old children in the studies of Hillman et al. (2009), Drollette et al. (2012), Gallotta et al. (2012), Chen et al. (2014), and Janssen et al. (2014a). In this respect, a recent meta-analysis found the largest effects of acute aerobic exercise bouts on reaction time measures of executive functioning in preadolescent children (6–12 years), as compared to older adolescents (13–19 years) (Ludyga et al., 2016).

Working memory performance was neither affected by exercise bouts of 10, 20, or 30 min, which is in line with previous studies that used a similar n-back task (Drollette et al., 2012; Soga et al., 2015), as well as studies that used a Sternberg task (Cooper et al., 2013) or a mixed dot task (Kubesh et al., 2009). In contrast to our results, a study of Chen et al. (2014) found faster reaction times after exercising compared to the control session on a n-back task (Chen et al., 2014). In their study, only the 2-back load of the n-back was used and their stimuli remained on screen for a considerably longer time than in our study (i.e., 2,000 vs. 500 ms). Hence, the n-back version we used in our study could be considered as more difficult due to a shorter memory trace. The difficulty of the n-back version we used in the current study could have contributed to the fact we were not able to detect the subtle effects of exercise on working memory performance. There were also differences in age and exercise activities (8–11 years old children and group-based running exercises in Chen et al., 2014), but not in the sample size tested (*N* = 92 in our study and *N* = 98 in Chen et al., 2014). The n-back task is often used to train cognitive performance, as it engages multiple executive functions at once which allows little room for employing automatic processes or task-specific strategies to optimally perform this task (Jaeggi et al., 2008). In retrospect, the n-back task is highly susceptible to intra-individual differences and might not be the best choice to investigate the subtle effects of exercise on performance.

Another purpose of our study was to assess the possible dose-response effects of 10, 20, and 30 min of moderate to vigorous intensity exercise on cognitive performance. As we found no acute effects of any exercise duration, we cannot make statements on a “longer duration—better performance” type of function between exercise and cognitive performance. This is in line with the study of Howie and colleagues who found no improvement in executive functioning after exercise bouts of either 5, 10, or 20 min (Howie et al., 2015). In contrast, they did report higher math fluency scores after 10 and 20 min of exercise compared to a 10-min sedentary control condition (Howie et al., 2015). However, the math fluency test measures other domains of cognitive/academic performance and is therefore difficult to compare with our findings. Although we recruited~15% more participants than needed based on the sample size calculation (*n* = 105 to detect differential effects of exercise duration), the final number of adolescents included in the data analyses was 92 in the n-back task and 99 in the ANT analyses. However, given the small effect sizes and large p-values that we found (η^2^ = 0.002–0.017 and *p* = 0.463–0.914), we expect that the lack of significant effects is not due to lack of power. We measured cognitive performance immediately before and after our 10-, 20-, and 30-min condition. Although we found no differences related to the time between pre- and posttest across the three conditions, further research on dose-response effects should consider to use comparable time frames between testing (e.g., group A: sitting for 20 min followed by 10 min of exercise, group B: sitting for 10 min followed by 20 min of exercise, and group C: exercising for 30 min).

Although we found no positive acute effects of exercise on selective attention and working memory measured immediately after the exercise bouts, adolescent's performance did not deteriorate after exercising for 10, 20, or 30 min compared to working on school related tasks. This is in line with the (sub)conclusions of several recently published systematic reviews and meta-analyses on the acute effects of exercise on cognition in children and adolescents (e.g., Donnelly et al., 2016; Li et al., 2017; Daly-Smith et al., 2018; de Greeff et al., 2018). Hence, implementing single exercise bouts of moderate to vigorous intensity throughout the school day does not seem to harm cognitive performance, and may help to increase the overall physical activity levels of children and adolescents (WHO, 2011; Bassett et al., 2013). The relevance of implementing exercise bouts for academic achievement in the long term needs further study. Therefore, we recommend researchers to investigate whether the longer term implementation of single exercise bouts may result in improved cognitive and academic performance of children and adolescents. Repeated exercise bouts may also increase enjoyment of school lessons and thereby improve cognitive and academic performance.

## Conclusion

In summary, acute moderate to vigorous exercise bouts with a duration of 10, 20, and 30 min did not improve nor deteriorate selective attention and working memory performance of young adolescents immediately after exercising, compared to a control condition in which they worked on school-related tasks. We found no differential effects of exercise bouts of relatively long, medium, and short duration.

## Author contributions

VvdB, ES, JJ, RdG, MC, and AS conceived and designed the study; VvdB and ES performed the data acquisition and analyzed the data; VvdB, JJ, RdG, MC, and AS contributed to the data interpretation and presentation; ES wrote the initial manuscript; VvdB, JJ, RdG, MC, and AS made critical revisions on several drafts of the manuscript; VvdB revised the initial manuscript and wrote the final version. All authors approved the final version of the manuscript.

### Conflict of interest statement

The authors declare that the research was conducted in the absence of any commercial or financial relationships that could be construed as a potential conflict of interest.
